# Urinary Polycyclic Aromatic Hydrocarbon Metabolites and Attention/Deficit Hyperactivity Disorder, Learning Disability, and Special Education in U.S. Children Aged 6 to 15

**DOI:** 10.1155/2014/628508

**Published:** 2014-01-30

**Authors:** Zaynah Abid, Ananya Roy, Julie B. Herbstman, Adrienne S. Ettinger

**Affiliations:** ^1^Department of Environmental Health Sciences, Yale School of Public Health, New Haven, CT 06520, USA; ^2^Department of Chronic Disease Epidemiology, Yale School Public Health, New Haven, CT 06520, USA; ^3^Department of Environmental Health Sciences, Columbia Mailman School of Public Health, New York, NY 10032, USA; ^4^Yale Center for Perinatal, Pediatric, and Environmental Epidemiology, 1 Church Street, 6th floor, New Haven, CT 06510, USA

## Abstract

Exposure to polycyclic aromatic hydrocarbons (PAHs) adversely affects child neurodevelopment, but little is known about the relationship between PAHs and clinically significant developmental disorders. We examined the relationship between childhood measures of PAH exposure and prevalence of attention deficit/hyperactivity disorder (ADHD), learning disability (LD), and special education (SE) in a nationally representative sample of 1,257 U.S. children 6–15 years of age. Data were obtained from the National Health and Nutrition Examination Survey (NHANES) 2001–2004. PAH exposure was measured by urinary metabolite concentrations. Outcomes were defined by parental report of (1) ever doctor-diagnosed ADHD, (2) ever doctor- or school representative-identified LD, and (3) receipt of SE or early intervention services. Multivariate logistic regression accounting for survey sampling was used to determine the associations between PAH metabolites and ADHD, LD, and SE. Children exposed to higher levels of fluorine metabolites had a 2-fold increased odds (95% C.I. 1.1, 3.8) of SE, and this association was more apparent in males (OR 2.3; 95% C.I. 1.2, 4.1) than in females (OR 1.8; 95% C.I. 0.6, 5.4). No other consistent pattern of developmental disorders was associated with urinary PAH metabolites. However, concurrent exposure to PAH fluorine metabolites may increase use of special education services among U.S. children.

## 1. Introduction

Polycyclic aromatic hydrocarbons (PAHs) are a class of ubiquitous environmental contaminants formed by incomplete combustion of organic material. The main sources of human exposure include occupation, passive and active smoking, food, water, and ambient air pollution [1]. Children may face increased vulnerability to environmental exposures, including PAHs, due to their unique behavior patterns and higher ingestion and inhalation rates given their body size. In a representative sample of the U.S. population, children (aged 6–11 years) had higher levels of PAH urinary metabolites than adolescents (aged 12–19) and adults (aged ≥20 years) [2]. This is consistent with previous studies of urinary PAH metabolites in children [3, 4] and, also is consistent with children's higher PAH intakes from diet, air, and soil [5, 6].

At least five PAH compounds, including benzo(a)pyrene, are considered by the EPA and IARC to be “probable” or “possible” human carcinogens via a genotoxic mechanism [7]. Animal studies have shown that prenatal PAH exposure may cause adverse neurodevelopment, including learning deficits, memory impairments, and behavioral changes [8–10]. The developing brain is particularly vulnerable to these effects and molecular epidemiological studies found that newborns may be up to 10 times more susceptible to DNA-PAH adduct formation than their mothers [11, 12]. The mechanisms by which PAH exposure affects a child's developing brain are not fully understood, but proposed mechanisms include endocrine disruption [13], oxidative stress [8], binding to placental growth factor receptors [14], induction of P450 enzymes via binding to the human Ah receptor [15], DNA damage resulting in activation of apoptotic pathways [16, 17], or epigenetic effects [18].

Prenatal exposure to PAHs predicted lower mental development index and cognitive developmental delay in the first three years of life [19]; symptoms of anxious/depressed and attention problems at age 5–7 years [20, 21]; decreased language, social, and average developmental quotients in 2-year-old children [22]; and decreased IQ at age of 5 [23, 24]. Higher referral by teachers for clinical assessment of learning and behavioral problems has also been reported [25].

Attention-deficit/hyperactivity disorder (ADHD) is a commonly recognized behavioral disorder characterized by symptoms of inattention and/or impulsivity. ADHD is believed to have genetic origins, but it may interact with the environment such that environmental factors act as a trigger for the disorder [26, 27]. Learning disability (LD) is often associated with ADHD, having been reported in 70% of ADHD patients and their relatives [28]. More than half of children with ADHD are also school-identified as eligible for SE services under the Individuals with Disabilities Education Act (IDEA), with most falling under the categories of LD, emotional disturbance, or other health impairment [28]. A study of elementary school children receiving special education (SE) services revealed that 44% of the children met the diagnostic criteria for ADHD, while only half of those were receiving care for the disorder [29]. Weiland et al. [30] estimated annual costs of preschool SE services for low-income NYC children with PAH-induced developmental delay to be $13.7 million per birth cohort.

There have been no reports of associations between children's PAH exposure and clinically significant neurodevelopmental effects in a nationally representative sample. Here, we assess the cross-sectional relationship between concurrent levels of urinary PAH metabolites and ADHD, LD, and SE in a nationally representative sample of U.S. children aged 6 to 15 years.

## 2. Materials and Methods

### 2.1. Data Source

Data were obtained from the 2001-2002 and 2003-2004 cycles of the National Health and Nutrition Examination Survey (NHANES) for children 6–15 years of age. NHANES is a nationally representative, cross-sectional survey of the noninstitutionalized U.S. civilian population, based on a complex, multistage probability sample. Details of the NHANES protocol are described elsewhere [31, 32]. Briefly, one to two weeks after an initial in-person interview, participants undergo a physical examination in specially designed and equipped Mobile Examination Centers (MECs). The laboratory component involves the collection of biological specimens, including urine for subjects aged 6 years and older.

In 2001–2004, laboratory analysis of urinary biomarkers was performed for one-third of participants aged 6 years and over. Monohydroxy-PAH (OH-PAH) metabolites were measured by an analytical method involving enzymatic hydrolysis of urine, extraction, derivatization, and analysis by capillary gas chromatography combined with isotope-dilution high-resolution mass spectrometry (GC-HRMS) [33].

### 2.2. PAH Exposure

Urinary metabolite concentrations (nanograms per liter (ng/L)) of eight PAHs, available in both the 2001-2002 and 2003-2004 cycles, were identified and selected for the data analysis: 1-pyrene, 1-napthol, 2-napthol, 2-fluorene, 3-fluorene, 1-phenanthrene, 2-phenanthrene, and 3-phenanthrene. Analytes below the limit of detection (LOD) were assigned a value of the LOD divided by the square root of 2.

Five exposure variables were used in this analysis: 1-pyrene, 1-napthol, 2-napthol, fluorine “FLUO” metabolites (2-fluorene + 3-fluorene), and phenanthrene “PHEN” metabolites (1-phenanthrene + 2-phenanthrene + 3-phenanthrene). Urinary 1-pyrene is the most commonly utilized PAH biomarker of exposure and is a useful surrogate of total PAH exposure [2]. The metabolites that arise from the same parent compound were combined, so that our exposure variables would more closely reflect environmental exposure to the parent compound. 1-napthol and 2-napthol were not combined, however, because these metabolites may reflect exposures to different source of naphthalene (personal communication, Dr. Andreas Sjödin, PAH Biomarker Laboratory, CDC). Since the ranges of PAH concentrations were positively skewed, the five exposure variables were log-transformed and treated as continuous variables and, in separate analyses, high/low categorical PAH variables, dichotomized at the median, were used.

### 2.3. Outcomes

The primary outcome of interest was parental report of ever-doctor-diagnosed ADHD. The survey question, as asked to participants, was “has a doctor or health professional ever told {you/survey  participant  (SP)} that {s/he/SP} had attention deficit disorder?” Secondary outcomes were parental report of LD and receipt of special education or early intervention services (abbreviated as “SE” throughout). The survey questions were as follows: “has a representative from a school or a health professional ever told {you/SP} that {s/he/SP} had a learning disability?” and “does {SP} receive special education or early intervention services?”

### 2.4. Covariates

A number of covariates and potential confounders identified in the literature were investigated, including age, sex, race/ethnicity (non-Hispanic white, non-Hispanic black, Hispanic and other), and body mass index (BMI) (<25, 25–30, and >30). Because urinary PAH metabolite concentrations vary based on urine dilution, log-transformed creatinine concentration was included as a covariate. The poverty-income ratio (PIR) (<1.00, 1–1.84, 1.85–3, >3), a ratio of family income to the poverty threshold, was used as an indicator of socioeconomic status. Health insurance coverage and having a routine health care provider, both of which have been associated with an increased likelihood of an ADHD diagnosis [34], were also included. Birth and early childhood factors including birthweight (<5.5 and ≥5.5 lbs), maternal age at birth, treatment at a newborn care facility, and preschool attendance were evaluated. Maternal smoking during pregnancy (yes/no) and having any smokers in the household (yes/no) were positively correlated (Spearman correlation coefficient = 0.344, *P* < 0.001); therefore, the “smokers in the household” variable was chosen for inclusion into the multivariate models to represent postnatal environmental tobacco smoke (ETS) given that we are evaluating the effect of concurrent PAH levels, rather than prenatal PAH levels. Log-transformed fasting time (hours since last meal) was also evaluated as a covariate since recent consumption of dietary PAHs may influence urinary metabolite concentrations.

### 2.5. Statistical Analysis

All analyses were performed using SAS v. 9.3 (SAS Institute Inc. Cary, NC), using SAS SURVEY procedures to account for the complex survey design of the NHANES sample. Variables found to be associated with each dichotomous yes/no outcome of interest based on a Chi-square test (categorical) or *t*-test (continuous) using a statistical threshold of *P* < 0.2 in bivariate analyses were included in the multivariate logistic regression analysis. Non-significant covariates (*P* > 0.05) were removed one at a time (backward elimination) as long as effect estimate between PAH exposure and outcome was not altered by greater than 10% in order to arrive at most parsimonious model. Regardless of statistical significance, the final models included age, sex, race/ethnicity, and creatinine, since PAH levels will vary according to urinary dilution, and urinary dilution varies by age, sex, and race/ethnicity group. To assess potential effect modification by sex, the interaction term sex ∗ PAH for each PAH exposure variable was included in modeling, and subgroup analyses were also performed. Because the initially selected covariates for each outcomes were nested within the larger group of covariates selected in the SE model and the results did not change appreciably (data not shown), the covariates found to be significant in the SE model were also used in the final models for ADHD and LD in order to achieve consistency among the three outcomes. Thus, the final models included adjustment for age, sex, race/ethnicity, creatinine, smokers in the household, PIR, birthweight, and having a routine source of health care. The sample was restricted to participants with no missing data for the PAH measures, covariates, or outcomes of interest.

## 3. Results

Table 1 shows the concentrations of the eight urinary PAH metabolites in the 2001-2002 and 2003-2004 NHANES participants aged 6–15 with no missing covariates or outcomes (*N* = 1257). Median urinary PAH metabolite concentrations (ng/L) were 1-pyrene, 76.1; 1-napthol, 1290.1; 2-napthol, 2036.6; 2-fluorene, 254.3; 3-fluorene, 102.2; 1-phenanthrene, 128.7; 2-phenanthrene, 49.1; 3-phenanthrene, 106.0. No influential outliers of the continuous covariates were observed and, thus, no observations were excluded prior to analysis.

Prevalences of ADHD, LD, and SE were 7.1%, 13.2%, and 11.4%, respectively, and the Spearman correlation coefficients were for ADHD and LD, *ρ* = 0.344 (*P* < 0.001); for ADHD and SE, *ρ* = 0.301 (*P* < 0.001); and for LD and SE, *ρ* = 0.679 (*P* < 0.001). Spearman correlation coefficients between PAH exposure variables and having any smokers in the household were all statistically significant (*P* < 0.05) and ranged from *ρ* = 0.056 for log-transformed 1-napthol to *ρ* = 0.437 for high/low FLUO metabolites. The dichotomous high/low exposure variables generally demonstrated a higher correlation coefficient with the “smokers in the household” variable than did the log-transformed continuous variables.

Bivariate analyses showed significant associations between sex, having smokers in the household, and maternal smoking during pregnancy with ADHD (Table 2). Additionally, race/ethnicity, birthweight, PIR, and fasting time were significant bivariate predictors for LD. All of those variables, as well as age and having a routine source of medical care, were significant bivariate predictors of SE.

Multivariate analyses resulted in a significant inverse association between log-transformed (continuous) 2-napthol and ADHD (OR = 0.75; 95% C.I. 0.59, 0.94) (Table 3). Smokers in the household and sex remained significant, independent predictors of ADHD in the final models for each of the continuous and categorical exposure variables (data not shown). In models stratified by sex, a significant inverse association was found between log-transformed 1-pyrene and ADHD (OR = 0.46; 95% C.I. 0.23, 0.94) and between high 2-napthol and ADHD (OR = 0.39; 95% C.I. 0.16, 0.98) among females. The interaction terms of PAH exposure variables with sex were all nonsignificant.

Overall, there were no significant associations between PAH metabolites and LD in multivariate analyses (Table 4). Smokers in the household, birthweight, sex, poverty, and race/ethnicity (higher risk for Hispanics and “other” race compared to whites) remained significant, independent predictors of LD in the final models for each of the continuous and categorical exposure variables (data not shown). However, in models stratified by sex, a significant inverse association was found between log-transformed 1-pyrene and LD (OR = 0.58; 95% C.I. 0.34, 0.98) (Table 4) among females.

Conversely, a marginally significant positive association between high 1-pyrene and SE (OR = 1.83; 95% C.I. 1.00, 3.34) was observed among males in multivariate adjusted models (Table 5). Additionally, high fluorine metabolite levels were significantly and positively associated with SE (OR = 2.00; 95% C.I. 1.05, 3.84). Smokers in the household, birthweight, male sex, poverty, and having a routine source of care remained significant, independent predictors of SE in the final models for each of the continuous and categorical exposure variables (data not shown). In models stratified by sex, a significant positive association between high fluorine and SE remained among males (OR = 2.25; 95% C.I. 1.24, 4.07), but it was not statistically significant among females (OR = 1.78; 95% C.I. 0.59, 5.35).

## 4. Discussion

Previous studies which have examined neurotoxic effects of prenatal PAHs in children were of modest sample size and many were based in New York City [19–21, 24] and China [22] where the results pertaining to highly-exposed populations may not be applicable to the general U.S. population. This study evaluated the association between childhood PAH biomarkers and parental report of ever doctor-diagnosed ADHD, LD, and receipt of SE services in a large, nationally-representative sample of U.S. children.

Significant inverse associations were observed between log-transformed 2-napthol and ADHD. Additionally, sex-stratified analyses indicated significant inverse associations among females between 1-pyrene and both ADHD and LD and between high 2-napthol and ADHD.

Conversely, significant positive associations were observed between high fluorine metabolites and SE. When stratified by sex, this association remained statistically significant for males but not for females. These findings suggest that PAH exposure may increase or decrease the risks of certain developmental disorders depending on the child's sex and the specific compounds to which they are exposed.

A number of limitations in this study must be considered. Unlike previous research on neurotoxic effects of PAHs, NHANES data are collected cross-sectionally thereby limiting our ability to infer causality. Current PAH levels may not reflect past exposures, and if prenatal and early childhood periods alone are the critical windows for susceptibility to the effects of exposure, then the use of current PAH levels as surrogate measures of etiologically-relevant exposures may have biased our results. Despite our findings regarding specific metabolites, it is difficult to attribute health effects to specific PAHs because most exposures are to PAH mixtures and not to individual PAH compounds [7].

Furthermore, use of urinary PAH metabolites, which have half-lives as short as 6–35 hours, will not reflect long-term exposures [35]. PAH-DNA adducts, which do not depend solely on the magnitude of recent dose, are a better measure of chronic exposure. Urinary concentrations are also affected by time of day when spot urine samples are collected—the within-day variance of urinary PAHs contributes 72 to 89% to total variance among individuals—a fluctuation which may be caused by diet, metabolism, or changes in daily routine [36]. Taking urine measurements at multiple time points or over a 24-hour period could help correct for exposure misclassification, although concentrations of urinary PAH metabolites reflect metabolic/detoxification capacity which also will vary among individuals. Our primary outcome is limited by its reliance on parental report of doctor-diagnosed ADHD rather than confirmed diagnosis using the Diagnostic and Statistical Manual of Mental Disorders, Fourth Edition (DSM-IV) criteria, or the Diagnostic Interview Schedule for Children IV (DISC-IV) used in previous studies. Similarly, diagnosis of LD and receipt of SE, including early intervention services, were both assessed via parental report. However, maternal reports of ADHD have been previously found to have high reliability and accuracy [37]. Nonetheless, any misclassification of the outcome should not be related to PAH exposures and, therefore, would have biased results towards the null.

Adjustment for potential confounders was limited to variables available for both NHANES cycles. Although presence of smokers in the household was included as a measure of ETS exposure, the potential for residual confounding exists. We did not quantitatively adjust for multiple comparisons in our statistical tests, which may have resulted in one or more of the observed effects. Additionally, because our sample size was limited by age restrictions, rarity of outcomes, and the one-third subsample of PAH measurements, we may have had insufficient power to detect some of the independent effects.

It is possible, as we found with some of the exposure variables, that PAH exposure does not increase the risk of ADHD, LD, or receipt of SE services in the general U.S. child population when controlling for other known risk factors. This would be consistent with a recent finding that prenatal exposures to PAHs showed no significant association with DSM-oriented attention deficit/hyperactivity problems at age 6-7 years in the Columbia Center for Children's Environmental Health (CCCEH) cohort [21].

Finally, as we have shown here, the effects of PAH exposure may differ by sex. Our sex-specific findings suggest that males may be at higher risk than females of PAH-induced neurodevelopmental effects. Recent research implicates sex-specific epigenetic marks—triggered by environmental factors—as a potential cause of the sex bias observed in many common diseases [38]. Epigenetic malprogramming caused by environmental factors may occur in a sex-specific manner, thereby leading to dimorphic gene expression and increased sensitivity of males (or females) to environmentally induced adverse health outcomes. PAHs, in particular, have been shown to alter gene expression in hormonal regulatory pathways (that are crucial to early brain development), alter expression of proinflammatory cytokines, upregulate *COX-2* (an enzyme involved in inflammation and associated with reactive oxygen species) [39], and, in the CCCEH cohort, to lead to differential methylation in umbilical cord white blood cell DNA of newborns [40]. More research is needed to detect whether these or other epigenetic alterations mediate the neurodevelopmental effects of PAHs and whether these effects are sex-specific.

Nonetheless, this is the first study to examine the relationship between childhood PAH exposure and ADHD, LD, and SE in a nationally representative sample of U.S. children. Our results suggest an association between fluorine metabolites and increased need for SE services among the general U.S. population of school-aged children aged 6–15 years with possible sex-specific effects. Large, nationally representative, prospective studies are needed to fully investigate the neurodevelopmental effects of PAH exposure in children and to disentangle the effects of prenatal and postnatal exposures.

## Figures and Tables

**Table 1:** Urinary PAH metabolite concentrations (ng/L), NHANES 2001–2004 participants aged 6–15 with no missing covariates or outcomes (*N* = 1257).

PAH metabolite	Percent below LOD^a^	Minimum	Percentiles (95% confidence intervals)						Maximum
			5th	10th	25th	50th	75th	95th	
1-pyrene	0.5	2.1	15.3 (11.6, 18.9)	21.7 (18.6, 24.8)	37.9 (32.9, 42.9)	76.1 (65.0, 87.2)	161.0 (143.4, 178.6)	483.5 (61.6, 357.8)	2216
1-napthol	0.1	33	263.8 (93.5, 334.1)	404.1 (334.7, 473.5)	722.5 (648.2, 796.7)	1290.1 (1140.9, 1439.2)	2969.2 (2572.6, 3365.9)	11165 (9373.5, 12956.1)	567902
2-napthol	0	27	354.5 (269.8, 439.2)	619.3 (527.4, 711.1)	1063.9 (977.8, 1150.0)	2036.6 (1812.7, 2260.5)	3622.8 (3233.3, 4012.3)	9748.2 (7344.2, 12152.1)	345241
FLUO metabolites									
2-fluorene	0	4	52.1 (43.7, 60.4)	81.2 (66.7, 95.8)	141.8 (126.9, 156.8)	254.3 (228.0, 280.6)	395.9 (357.8, 434.0)	875.1 (772.8, 977.3)	7828.6
3-fluorene	0.1	3.5	22.3 (17.2, 27.5)	33.9 (28.1, 39.7)	60.9 (53.5, 68.3)	102.2 (94.7, 109.7)	167.0 (154.7, 179.2)	386.3 (322.8, 449.8)	3462.7
PHEN metabolites									
1-phenanthrene	0.3	2.8	30.0 (24.3, 35.8)	43.0 (36.3, 49.6)	74.8 (66.2, 83.5)	128.7 (116.2, 141.2)	225.1 (201.4, 248.8)	506.5 (407.5, 605.6)	2297
2-phenanthrene	4.1	2.1	5.8 (2.0, 9.6)	12.4 (8.9, 16.0)	24.9 (20.5, 29.3)	49.1 (43.9, 54.4)	82.9 (74.2, 91.7)	226.6 (195.2, 257.9)	2469
3-phenanthrene	0.6	2.8	22.9 (18.3, 27.4)	35.1 (30.3, 40.0)	61.3 (55.6, 66.9)	106.0 (94.0, 118.0)	186.8 (167.7, 205.8)	413.6 (315.1, 512.1)	19643

^a^Limits of detection (LOD) (ng/L) for PAH metabolites.

2001-2002: 1-pyrene: 3.3, 1-napthol: 6.2, 2-napthol: 2.4, 2-fluorene: 3.6, 3-fluorene: 2.0, 1-phenanthrene: 3.5, 2-phenanthrene: 3.2, and 3-phenanthrene: 3.6.

2003-2004: 1-pyrene: 4.9, 1-napthol: 18, 2-napthol: 12, 2-fluorene: 4.5, 3-fluorene: 6.9, 1-phenanthrene: 2.6, 2-phenanthrene: 3.8, and 3-phenanthrene: 2.6.

**Table 2:** Descriptive characteristics by ADHD, LD, and SE, NHANES 2001–2004 participants aged 6–15 with no missing covariates or outcomes (*N* = 1257)^a^.

Characteristic	Ever diagnosed with ADHD			Ever diagnosed with LD			Receiving SE		
	Yes	No	*P* ^c^	Yes	No	*P* ^c^	Yes	No	*P* ^c^
	(*N* = 83)^b^	(*N* = 1174)^b^		(*N* = 147)^b^	(*N* = 1113)^b^		(*N* = 129)^b^	(*N* = 1128)^b^	
Age (years)	11.2 ± 0.5	11.0 ± 0.4	0.222	11.0 ± 0.4	10.6 ± 0.2	0.26	11.3 ± 0.3	10.6 ± 0.2	0.017
Sex			0.002			0.002			0.003
Male	58 (5.3)	550 (44.3)		89 (7.6)	519 (42.0)		84 (7.0)	524 (42.6)	
Female	25 (2.2)	624 (48.2)		57 (4.2)	592 (46.2)		45 (3.7)	604 (46.7)	
Race/ethnicity			0.545			0.019			0.06
White	31 (5.1)	319 (56.1)		46 (7.9)	304 (53.4)		41 (7.1)	309 (54.1)	
Black	29 (1.0)	409 (14.3)		64 (2.3)	374 (13.1)		53 (2.0)	385 (13.4)	
Hispanic	21 (1.2)	410 (17.2)		35 (1.5)	396 (16.9)		34 (1.4)	397 (17.0)	
Other	2 (0.2)	36 (4.8)		1 (0.2)	37 (4.9)		37 (4.9)	1 (0.2)	
Birthweight			0.584			0.003			<0.001
≥5.5 lbs	69 (6.5)	1018 (82.4)		115 (9.5)	972 (79.4)		97 (8.2)	990 (80.8)	
<5.5 lbs	14 (1.0)	156 (10.0)		31 (2.3)	139 (8.7)		32 (2.5)	138 (8.6)	
Received newborn care at health facility			0.912			0.386			0.645
Yes	12 (1.0)	145 (12.1)		26 (1.9)	131 (11.2)		23 (1.6)	134 (11.5)	
No	71 (6.6)	1029 (80.3)		120 (9.9)	980 (77.0)		106 (9.1)	994 (77.8)	
Maternal age at birth (years)	26.4 ± 0.9	25.7 ± 0.6	0.985	25.7 ± 0.6	26.5 ± 0.3	0.198	25.6 ± 0.6	26.5 ± 0.3	0.169
Maternal smoking during pregnancy			0.007			<0.001			<0.001
Yes	27 (2.9)	173 (18.1)		43 (4.6)	157 (16.4)		44 (4.6)	156 (16.5)	
No	56 (4.7)	1001 (74.3)		103 (7.2)	954 (71.8)		85 (6.1)	972 (72.9)	
Smoker(s) in household			0.013			<0.001			0.003
Yes	26 (2.9)	242 (19.8)		54 (4.7)	214 (18.0)		43 (4.0)	225 (18.7)	
No	57 (4.6)	932 (72.7)		92 (7.1)	897 (70.2)		86 (6.7)	903 (70.6)	
Has routine source of care			0.691			0.485			<0.001
Yes	81 (7.2)	1071 (87.1)		138 (11.3)	1014 (83.0)		126 (10.6)	1026 (83.7)	
No	2 (0.3)	81 (7.2)		8 (0.5)	97 (5.2)		3 (0.1)	102 (5.6)	
Health insurance coverage			0.221			0.787			0.097
Yes	77 (7.0)	981 (80.5)		129 (10.4)	929 (77.0)		118 (9.8)	940 (77.6)	
No	6 (0.6)	193 (12.0)		17 (1.4)	182 (11.2)		11 (0.8)	188 (11.7)	
BMI			0.668			0.255			0.365
<25	70 (6.6)	944 (78.0)		108 (9.3)	906 (75.3)		101 (8.7)	913 (75.9)	
25–30	9 (0.5)	152 (9.8)		23 (1.6)	138 (8.7)		17 (1.1)	144 (9.2)	
>30	4 (4.7)	78 (4.7)		15 (0.9)	67 (4.2)		11 (0.9)	71 (4.2)	
Ever attended preschool			0.695			0.654			0.735
Yes	66 (5.4)	778 (64.1)		104 (8.5)	740 (61.1)		91 (7.6)	753 (61.9)	
No	17 (2.1)	396 (28.3)		42 (3.4)	371 (27.1)		38 (3.1)	375 (27.4)	
PIR			0.113			0.008			0.028
<1	26 (2.8)	392 (20.3)		64 (4.6)	354 (19.0)		57 (4.1)	361 (19.5)	
1–1.84	18 (1.3)	266 (17.8)		32 (2.2)	252 (17.0)		25 (1.6)	259 (17.6)	
1.85–3	17 (1.1)	218 (19.1)		28 (2.3)	207 (17.9)		26 (2.3)	209 (17.9)	
>3	22 (2.3)	298 (34.7)		22 (2.7)	298 (34.3)		21 (2.7)	299 (34.3)	
Creatinine mg/dL (log-transformed)	4.5 ± 0.1	4.6 ± 0.0	0.4	4.6 ± 0.1	4.6 ± 0.0	0.867	4.7 ± 0.1	4.6 ± 0.0	0.809
Fasting time hr (log-transformed)	1.9 ± 0.1	1.9 ± 0.1	0.316	2.0 ± 0.1	1.7 ± 0.1	0.023	2.0 ± 0.1	1.7 ± 0.1	0.037
1-Pyrene ng/L (log-transformed)	4.3 ± 0.2	4.4 ± 0.1	0.591	4.4 ± 0.1	4.4 ± 0.1	0.826	4.5 ± 0.1	4.4 ± 0.1	0.288
1-Pyrene			0.785			0.819			0.087
High	46 (4.0)	580 (46.1)		76 (6.1)	550 (44.0)		74 (6.5)	552 (43.6)	
Low	37 (3.6)	594 (46.3)		70 (5.8)	561 (44.1)		55 (4.2)	576 (45.7)	
	7.2 ± 0.2	7.3 ± 0.0	0.702	7.4 ± 0.1	7.3 ± 0.0	0.25	7.4 ± 0.1	7.3 ± 0.1	0.335
1-Napthol			0.645			0.024			0.146
High	42 (3.6)	610 (46.4)		90 (7.1)	562 (42.9)		74 (6.1)	578 (44.0)	
Low	41 (3.9)	564 (46.0)		56 (4.7)	549 (45.3)		55 (4.6)	550 (45.4)	
2-Napthol ng/L (log-transformed)	7.3 ± 0.2	7.6 ± 0.0	0.14	7.6 ± 0.1	7.6 ± 0.0	0.856	7.8 ± 0.2	7.6 ± 0.0	0.16
2-Napthol			0.082			0.384			0.693
High	40 (2.9)	655 (47.2)		80 (5.3)	615 (44.8)		69 (5.6)	626 (44.5)	
Low	43 (4.7)	519 (45.3)		66 (6.5)	496 (43.4)		60 (5.1)	502 (44.8)	

^a^Table values are mean ± SD for continuous variables and *n* (%) for categorical variables.

^b^Percentages may not sum to 100% due to rounding.

^c^ *P* value is for *t*-test (continuous variables) or *χ* ^2^ test (categorical variables).

**Table 3:** Multivariate associations^a^ between PAH metabolites and ADHD, NHANES 2001–2004 (*N* = 1257).

	All (*N* = 1257) OR (95% CI)	Male (*N* = 608) OR (95% CI)	Female (*N* = 649) OR (95% CI)	*P* value for interaction^b^
1-Pyrene				
Log-transformed	0.91 (0.69, 1.21)	1.13 (0.88, 1.46)	0.46 (0.23, 0.94)	0.2
High versus low	1.22 (0.65, 2.28)	1.39 (0.76, 2.55)	0.90 (0.28, 2.88)	0.7
1-Napthol				
Log-transformed	1.00 (0.83, 1.21)	1.10 (0.87, 1.38)	0.85 (0.55, 1.31)	0.5
High versus low	1.00 (0.60, 1.69)	1.28 (0.62, 2.61)	0.63 (0.22, 1.81)	0.4
2-Napthol				
Log-transformed	0.75 (0.59, 0.94)	0.76 (0.58, 0.99)	0.78 (0.50, 1.20)	0.8
High versus low	0.59 (0.35, 1.01)	0.75 (0.44, 1.30)	0.39 (0.16, 0.98)	0.5
FLUO metabolites				
Log-transformed	0.93 (0.70, 1.24)	0.98 (0.69, 1.41)	0.84 (0.42, 1.70)	0.7
High versus low	1.40 (0.72, 2.72)	1.50 (0.68, 3.31)	1.22 (0.42, 3.53)	0.6
PHEN metabolites				
Log-transformed	0.93 (0.61, 1.42)	1.07 (0.75, 1.52)	0.68 (0.27, 1.74)	0.9
High versus low	0.71 (0.36, 1.39)	0.64 (0.33, 1.25)	0.87 (0.23, 3.20)	0.9

^a^Adjusted for age, race/ethnicity, sex, creatinine, smokers in the household, PIR, birthweight, and having a routine source of medical care.

^b^ *P* value for interaction term sex ∗ PAH.

**Table 4:** Multivariate associations^a^ between PAH metabolites and LD, NHANES 2001–2004 (*N* = 1257).

	All (*N* = 1257) OR (95% CI)	Male (*N* = 608) OR (95% CI)	Female (*N* = 649) OR (95% CI)	*P* value for interaction^b^
1-Pyrene				
Log-transformed	0.85 (0.68, 1.05)	0.99 (0.77, 1.27)	0.58 (0.34, 0.98)	0.8
High versus low	0.87 (0.50, 1.51)	1.28 (0.68, 2.41)	0.42 (0.16, 1.11)	0.4
1-Napthol				
Log-transformed	1.03 (0.85, 1.23)	1.01 (0.75, 1.35)	1.19 (0.84, 1.68)	0.3
High versus low	1.65 (0.96, 2.81)	1.81 (0.83, 3.93)	1.46 (0.64, 3.31)	0.9
2-Napthol				
Log-transformed	0.91 (0.71, 1.17)	1.00 (0.68, 1.46)	0.89 (0.54, 1.45)	0.5
High versus low	0.63 (0.31, 1.28)	0.75 (0.32, 1.76)	0.50 (0.18, 1.41)	0.9
FLUO metabolites				
Log-transformed	0.89 (0.68, 1.17)	0.67 (0.42, 1.08)	1.50 (0.80, 2.81)	0.2
High versus low	1.14 (0.58, 2.23)	0.93 (0.46, 1.89)	1.54 (0.56, 4.24)	0.3
PHEN metabolites				
Log-transformed	0.91 (0.67, 1.24)	0.90 (0.57, 1.42)	1.03 (0.49, 2.13)	0.1
High versus low	0.74 (0.42, 1.32)	0.69 (0.33, 1.44)	0.76 (0.29, 2.005)	0.1

^a^Adjusted for age, race/ethnicity, sex, creatinine, smokers in the household, PIR, birthweight, and having a routine source of medical care.

^b^ *P* value for interaction term sex ∗ PAH.

**Table 5:** Multivariate associations^a^ between PAH metabolites and SE, NHANES 2001–2004 (*N* = 1257).

	All (*N* = 1257) OR (95% CI)	Male (*N* = 608) OR (95% CI)	Female (*N* = 649) OR (95% CI)	*P* value for interaction^b^
1-Pyrene				
Log-transformed	1.10 (0.86, 1.42)	1.24 (0.94, 1.63)	0.82 (0.49, 1.38)	0.3
High versus low	1.52 (0.86, 2.68)	1.83 (1.00, 3.34)	1.07 (0.43, 2.66)	0.8
1-Napthol				
Log-transformed	1.05 (0.88, 1.26)	1.02 (0.77, 1.37)	1.17 (0.81, 1.69)	0.1
High versus low	1.32 (0.77, 2.25)	1.55 (0.73, 3.29)	1.01 (0.41, 2.51)	0.3
2-Napthol				
Log-transformed	1.18 (0.89, 1.57)	1.27 (0.88, 1.82)	1.06 (0.71, 1.59)	0.5
High versus low	1.00 (0.53, 1.89)	1.27 (0.62, 2.60)	0.68 (0.27, 1.74)	0.9
FLUO metabolites				
Log-transformed	1.32 (0.92, 1.89)	1.24 (0.88, 1.75)	1.55 (0.79, 3.03)	0.4
High versus low	2.00 (1.05, 3.84)	2.25 (1.24, 4.07)	1.78 (0.59, 5.35)	0.2
PHEN metabolites				
Log-transformed	1.25 (0.89, 1.75)	1.25 (0.87, 1.80)	1.25 (0.67, 2.34)	0.2
High versus low	1.01 (0.61, 1.66)	1.01 (0.53, 1.91)	1.07 (0.44, 2.63)	0.7

^a^Adjusted for age, race/ethnicity, sex, creatinine, smokers in the household, PIR, birthweight, and having a routine source of medical care.

^b^ *P* value for interaction term sex ∗ PAH.
